# Different modes of evolution in males and females generate dichromatism in fairy-wrens (Maluridae)

**DOI:** 10.1002/ece3.686

**Published:** 2013-08-01

**Authors:** Allison E Johnson, J Jordan Price, Stephen Pruett-Jones

**Affiliations:** 1 Department of Ecology and Evolution, The University of ChicagoChicago, Illinois, 60637-1503; 2 Department of Biology, St. Mary's College of MarylandSt. Mary's City, Maryland, 20686-3001

**Keywords:** Fairy-wrens, Maluridae, plumage evolution, selection, sexual dimorphism

## Abstract

Sexual dichromatism in birds is often attributed to selection for elaboration in males. However, evolutionary changes in either sex can result in plumage differences between them, and such changes can result in either gains or losses of dimorphism. We reconstructed the evolution of plumage colors in both males and females of species in Maluridae, a family comprising the fairy-wrens (*Malurus*, *Clytomias*, *Sipodotus*), emu-wrens (*Stipiturus*), and grasswrens (*Amytornis*). Our results show that, across species, males and females differ in their patterns of color evolution. Male plumage has diverged at relatively steady rates, whereas female coloration has changed dramatically in some lineages and little in others. Accordingly, in comparisons against evolutionary models, plumage changes in males best fit a Brownian motion (BM) model, whereas plumage changes in females fit an Ornstein Uhlenbeck (OU) multioptimum model, with different adaptive peaks corresponding to distributions in either Australia or New Guinea. Levels of dichromatism were significantly associated with latitude, with greater dichromatism in more southerly taxa. Our results suggest that current patterns of plumage diversity in fairy-wrens are a product of evolutionary changes in both sexes, driven in part by environmental differences across the distribution of the family.

## Introduction

The evolution and diversity of coloration in birds has long interested evolutionary biologists (Darwin 1871; Cronin 1991; Andersson 1994). Both sexual dichromatism and monochromatism are common in birds, and at least 150 independent transitions between these states have occurred within the passerines alone (Price and Birch 1996; but see Eaton 2005). This suggests that plumage is subject to many different and fluctuating selection pressures. Dichromatism results when selection differs between the sexes, either for increased or decreased ornamentation in either or both males and females (Cunningham and Birkhead 1998; Kimball and Ligon 1999; Amundsen 2000; Badyaev and Hill 2003). In fact, dimorphism in any trait can result from changes in either sex away from a shared pattern (Irwin 1994; Burns 1998; Wiens 2001; Hofmann et al. 2008; Friedman et al. 2009; Price et al. 2009).

Although ecological factors (e.g., breeding latitude, predation) are known to influence the evolution of dichromatism (Bennett and Owens 2002; Badyaev and Hill 2003), the most widely accepted paradigm involves sexual selection for elaboration of plumage in males (Andersson 1994; Amundsen 2000). The assumption implicit in this hypothesis is that species with elaborate or bright males and relatively dull females evolved from an ancestral condition in which both males and females were dull in plumage. Nevertheless, plumage in females can show considerable variation among taxa, and female plumage coloration is often under strong selection (Björklund 1991; Irwin 1994; Martin and Badyaev 1996; Burns 1998; Cunningham and Birkhead 1998; Amundsen 2000; Hofmann et al. 2008; Friedman et al. 2009). In at least one genus of birds, the New World orioles (*Icterus*), evidence suggests that sexual dichromatism has resulted from the repeated loss of bright plumage in females rather than gain of bright plumage in males (Hofmann et al. 2008; Friedman et al. 2009). Even in cases of sexual monochromatism in which both males and females are brightly colored, such patterns can result from mutual selection by males and females (Jones and Hunter 1993, 1999), social selection on females to retain bright plumage or ornamentation (Irwin 1994; Amundsen 2000), or even as a genetically correlated response to selection on males (Price and Whalen 2009).

**Figure 1:**
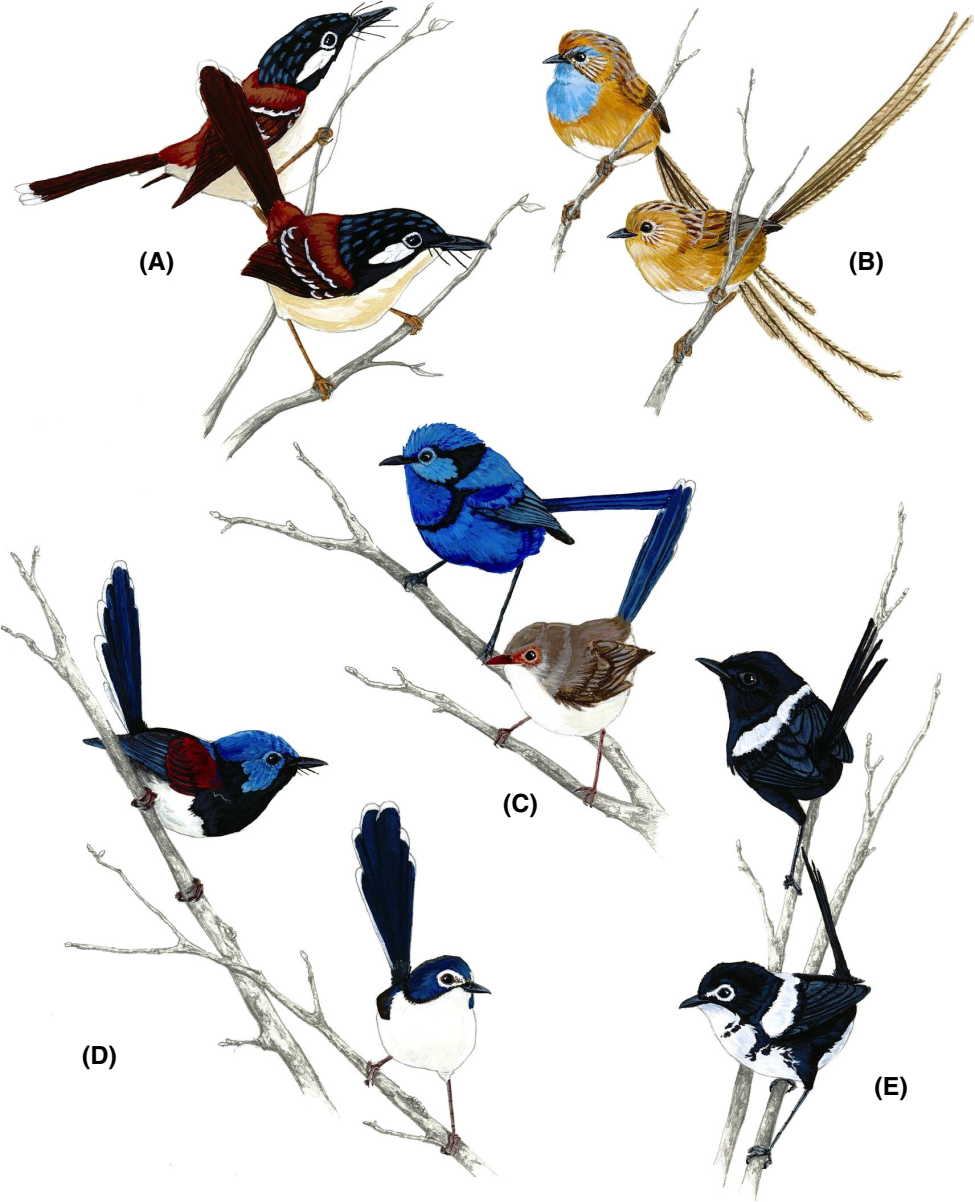
Illustration of both males and females of five species representative of the Maluridae, showing the range of plumage dimorphism present in the family: (A) Wallace's fairy-wren (*Sipodotus wallacii*), (B) Southern emu-wren (*Stipiturus malachurus*), (C) splendid fairy-wren (*Malurus splendens*), (D) lovely fairy-wren (*Malurus amabilis*), and (E) white-shouldered fairy-wren (*Malurus alboscapulatus*). Males are illustrated above females in each species pair.

In this study, we examine plumage evolution in the Maluridae, a relatively small (25 species) but diverse family of birds, comprising the fairy-wrens (*Malurus*, *Clytomias*, *Sipodotus*), grasswrens (*Amytornis*), and emu-wrens (*Stiptiturus*) (see Fig. 1). The Maluridae are endemic to Australia and New Guinea, and they evolved in Australasia as part of the ancient songbird radiation (Sibley and Ahlquist 1985, 1990; Christidis and Schodde 1997; Rowley and Russell 1997). Species occur in a wide range of habitats, from primary rainforest in New Guinea to desert grasslands in Australia, and in any geographical area as few as one species or as many as five species may occur. Plumage varies considerably both across and within species (Rowley and Russell 1997; Driskell et al. 2002, 2010; Karubian 2002), and levels of dichromatism differ across species as well. Dichromatism can also differ across populations within one species (e.g., *M. alboscapulatus*). Sexual selection is known to be important in many species of fairy-wrens (Webster et al. 2007), but the extent to which sexual selection influences coloration is presently unknown.

Our focus in this study was on the 21 malurid taxa included in the recent phylogeny by Driskell et al. (2011). Our objectives were to compare the evolution of plumage colors in males and females and to examine how these evolutionary changes have contributed to the evolution of sexual dichromatism in this group.

## Methods

Our study used the molecular phylogeny and DNA sequence data published by Driskell et al. (2011), and both that study and ours follow the taxonomic nomenclature of Dickinson (2003) and Christidis and Boles (2008). The phylogeny of Driskell et al. (2011) supports the general classification of the Australian fairy-wrens (*Malurus*) into several commonly recognized “coloration groups” (Christidis and Schodde 1997): the “blue group,” consisting of *Malurus cyaneus* and *M. splendens* (Fig. 1C), the “bi-colored group,” consisting of *M. alboscapulatus* (Fig. 1E), *M. melanocephalus*, and *M. leucopterus*, and the “chestnut-shouldered group,” consisting of *M. elegans*, *M. lamberti*, *M. pulcherrimus*, and *M. amabilis* (Fig. 1D), with *M. grayi*, *M. cyanocephalus*, and *M. coronatus* forming additional distinct color groups (Rowley and Russell 2007).

### Plumage scoring

Our methods for choosing and scoring plumage characters followed methods used by Omland and Lanyon (2000) and Price and Whalen (2009). Our study included 22 taxa representing 16 species, including all the Australian fairy-wren taxa (genus *Malurus*), most of the New Guinea fairy-wren taxa (*Malurus*, *Clytomias*, and *Sipodotus*) (see Fig. 1A for a representative species), one emu-wren (*Stipiturus malachurus*, Fig. 1B), one grasswren (*Amytornis striatus*), and a honeyeater (*Acanthorhynchus tenuirostris*) as an outgroup. The latter two species were included as outgroup taxa in the phylogeny of Driskell et al. (2011).

Plumage patches were scored as discrete characters for adult males and females of each of the 22 taxa. Although spectrometric methods are available for measuring color (e.g., Eaton 2005; Hofmann et al. 2008), our phylogenetic analyses (see below) required that we score color as discrete character states rather than as continuous reflectance values. We first scored plumage colors based on the illustrations in Rowley and Russell (1997). These scores were checked against illustrations in Schodde (1982). We then confirmed or revised these scores using museum skins from the collection at the Field Museum of Natural History (Chicago, IL) for males of 13 species and females of 12 species (57 specimens total). Except for one trait (coloration of the back), the scores we obtained from examination of the study skins were identical to those scores obtained from the illustrations in Schodde (1982) and Rowley and Russell (1997).

In all, we scored 25 color patches (body regions) and the presence/absence of five qualitative traits (30 characters total for both males and females; Tables 1A and 2A). We defined a color patch as a continuous region of the body with a similar coloration. If a body region (e.g., belly) exhibited color variation within it in any taxon, the region was split into two or more distinct patches (e.g., upper belly and lower belly) for all taxa. Colors were scored as either carotenoid (C), blue (B), black (N), dark brown (D), light brown (L), or white (W). As in the studies by Omland and Lanyon (2000) and Price and Whalen (2009), our color scores were discrete categories that each represented a continuous yet narrow range of colors. Scoring color in this way allowed us to identify discontinuous evolutionary changes in our phylogenetic reconstructions of plumage color evolution (Hofmann et al. 2008).

In some cases, different scores may reflect different underlying mechanisms of coloration, although the accuracy of such relationships likely had little impact on our reconstructions of evolutionary change. For example, the various shades of blue exhibited by many fairy-wrens (Rowley and Russell 1997) are all presumably the result of complex feather microstructure (Prum 2006) and so were combined in our analysis into one character state. Likewise, reds and oranges in birds are often products of carotenoid pigments (McGraw 2006a; but see McGraw et al. 2004), so we combined these colors as well. Blacks, dark browns, and light browns are generally due to melanin pigmentation in birds (McGraw 2006b); however, these colors differed enough consistently across our study taxa that we categorized them as different character states. Qualitative traits (e.g., whether an eye-line was present, or whether iridescence was prominent in the plumage) were scored as either present or absent.

Of the 30 characters (25 body regions and five presence/absence traits), 29 varied across taxa in males and 28 varied in females (Tables 2 and 3). Beak color was invariant in all males, whereas crown spot and eye-line were invariant in females. Across the 30 characters, the mean number of character states scored in males (3.73 ± 0.24) and in females (3.50 ± 0.27) across species did not differ significantly (paired *t*-test; *t*_29_ = 0.851; *P* = 0.402).

We measured dichromatism in each taxon as the percentage of color characters, of the 30 total, that differed between the sexes. While we determined percent dichromatism, we do not quantitatively or qualitatively describe any species as either monomorphic and bright or monomorphic and dull. Any mention of bright or dull species in the results or discussion is based on our perception of these species and typically we use “bright” when referring to nonbrown hues and “dull” as brown hues.

### Reconstruction of ancestral plumage states

We reconstructed ancestral states for male and female plumage characters separately on the molecular phylogeny of Driskell et al. (2011) using unordered parsimony in MacClade 4.08 (Maddison and Maddison 2005). Other methods of character reconstruction are available, such as maximum likelihood and Bayesian methods; however, our goal in this analysis was to identify statistically discontinuous evolutionary changes in each sex rather than the probability of particular ancestral states on the phylogeny. For each sex, we used the Trace All changes function in MacClade to count the number of unambiguous (i.e., unequivocal) changes on each branch of the tree. This method calculated the minimum number of changes on a branch by ignoring ambiguous cases in which ancestral changes were dependent on multiple, equally parsimonious states at a corresponding node. We included one of the outgroup taxa used by Driskell et al. (2011), *A. striatus*, in our analysis based on evidence that it is the sister taxon to the rest of the Maluridae (Lee et al. 2012). However, we did not include the nonmalurid outgroup taxon, *A. tenuirostris*.

To explore the effects of alternative phylogenetic hypotheses on our ancestral color reconstructions, we also reconstructed male and female color changes using relationships based on another recent molecular phylogeny of the Maluridae by Lee et al. (2012), which differs from that of Driskell et al. (2011) in the placement of *Malurus coronatus* as sister taxon to all the Australian *Malurus* rather than as sister taxon to the chestnut-shouldered fairy-wren group (*M. amabilis*, *M. lamberti*, *M. pulcherrimus*, and *M. elegans*). We compared the reconstructions using both phylogenies to assess how changes in the placement of *M. coronatus* affected our results.

We assessed the degree to which our plumage data were congruent with phylogeny by calculating the overall consistency index (CI) and overall retention index (RI) separately for males and females using MacClade. For both indices, a score of 1.0 indicates perfect congruence with phylogeny, with no evolutionary convergence or reversals, whereas a score approaching 0.0 indicates high levels of homoplasy. Calculating these scores allowed us to assess whether levels of evolutionary convergence in plumage differed between the sexes.

We further investigated evolutionary patterns in male and female coloration by plotting “plumage distance” between all possible pairs of taxa, including *A. tenuirostris*, as a function of molecular sequence divergence (uncorrected *p* distances), calculated using PAUP* 4.0 (Swofford 2002). We measured plumage distance as the number of plumage characters with different states, and we measured molecular divergence using sequence data from four mitochondrial genes (ND2, ND3, CO1, and ATP6) and three nuclear introns (FIB5, LDH, and GAPDH; sequences obtained from Driskell et al. 2011). We then calculated a linear regression through the points of each sex and calculated their coefficients of determination (*R*^2^). Although such pair-wise comparisons are not phylogenetically independent (Felsenstein 1985), we nevertheless felt that these relative values provided a useful means for comparing overall patterns of evolutionary change in plumage color between the sexes (also see Price and Whalen 2009).

### Testing evolutionary models

To examine whether plumage colors of males and females have evolved differently, we tested plumage patterns against two evolutionary models. The evolutionary models we considered were Brownian motion (BM) and single-optimum Ornstein Uhlenbeck (OU) (Butler and King 2004). BM models describe a “random walk,” and when applied to evolution can describe either selection randomly fluctuating through time or genetic drift (Felsenstein 1988; Harmon et al. 2010). OU models reflect natural selection toward a trait optimum, genetic drift on a highly constrained character, or genetic drift occurring on a character when stabilizing selection is weak and the adaptive peak is relatively close (Lande 1976; Felsenstein 1988). Although other models for character evolution exist (e.g., White Noise, Delta), we chose these two a posteriori based on the results of our analysis of plumage distance versus genetic divergence. Characters were considered unordered and unweighted, that is, all transitions were considered equally likely. All calculations and simulations were completed in R (R Development Core Team 2012), using the packages “vegan,” “geiger,” and “OUwie” (Harmon et al. 2009; Beaulieu and O'Meara 2012; Oksanen et al. 2012).

Although fairy-wrens occur in both Australia and New Guinea, the evolutionary history of the group appears to have involved multiple movements and subsequent radiations in different parts of their current distribution (Schodde 1982). It is also the case that Australia and New Guinea have repeatedly been connected by a land bridge, and thus movement from one region to the other may have been relatively easy. It has been hypothesized that the Maluridae first evolved in Australia (leading to today's emu-wren and grasswren clades), that the true fairy-wrens (the *Malurus* [*Chenorhamphus*] – *Sipodotus* – *Clytomyias* clades) evolved in New Guinea, and that fairy-wren (*Malurus*) taxa subsequently radiated on the mainland of Australia followed by a second movement to New Guinea by *M. alboscapulatus* (Schodde 1982) (for examples of each radiation see Fig. 1A–E, respectively). The *M. alboscapulatus* complex now includes six recognized subspecies that differ considerably in female plumage colors (Rowley and Russell 1997).

As Australia and New Guinea differ considerably in habitat (Australia being mostly arid and New Guinea being tropical), it seems likely that these differences could influence the evolution of plumage of bird species in each location. With this in mind, we further tested male and female plumage characters against two multioptimum OU models, treating hypothesized geographic origins of species as adaptive peaks. These models are similar to a single-optimum OU model in that they posit natural selection; however, multioptimum OU models allow for multiple adaptive peaks rather that a single adaptive peak. More specifically, our first multioptimum model had two adaptive peaks corresponding to location (Australia or New Guinea), and the second multioptimum model had four peaks corresponding to the four distinct distributions outlined above (1. Australian distribution of emu-wrens and grasswrens; 2. New Guinea distribution of the hypothesized basal fairy-wren taxa; 3. Australian species of *Malurus*; and 4. the New Guinea *M. alboscapulatus* complex). For each model we report log likelihood, Akaike weights, and Delta AIC (Akaike Information Criterion; Table 1). Delta AIC values are calculated from AIC values, with the lowest AIC score subtracted from each AIC score to produce the Delta AIC. Thus, the Delta AIC score with the value zero is the best fitting model. If this model is two or more points lower than other models, it is considered to have substantial empirical support as the best fitting model (Burnham and Anderson 2002). Additional model parameters are available upon request of the authors.

**Table 1:** Log-likelihood, delta AIC, and Akaike weight values from evolutionary models analysis for both male and female Maluridae species

	Brownian motion	Single-peak OU	2-Peak OU	4-Peak OU
Females				
Log-likelihood	−1.501	5.443	20.734	22.197
Delta AIC	38.749	27.563	0	4.322
Akaike weight	0	0	0.897	0.103
Males				
Log-likelihood	12.093	12.093	12.517	16.360
Delta AIC	0	2.702	4.874	4.435
Akaike weight	0.687	0.178	0.060	0.075

We also reconstructed these four ancestral distribution points as discrete character states on the phylogeny (Driskell et al. 2011). Locations were analyzed as discrete character states as the geographic regions are discrete and separated. This was done using the R package “ape” that employs maximum likelihood methods, similar to those methods employed by Mahler et al. (2010) in reconstructing geography to determine ancestral habitat (Paradis et al. 2004). Although there are other reconstruction methods for determining ancestral habitat (e.g., the software Lagrange; Ree and Smith 2008), given that we were using discrete characters, the use of alternative methods would not have altered our results.

### Relating dichromatism to latitude

To explore the relationship between latitude and sexual dichromatism, we performed a phylogenetically independent contrasts (PIC) analysis (Felsenstein 1985) using the PDAP:PDTREE module in Mesquite 2.75 (Maddison and Maddison 2011; Midford et al. 2011). This method incorporates phylogenetic relationships into comparisons of continuous variables among taxa to correct for statistical nonindependence due to shared history. We scored latitude (degrees south) as the mean between the upper and lower latitudinal limits of each species' range (Rowley and Russell 1997).

To ensure that the branch lengths of our phylogenetic tree adequately fit the tip data, we initially performed a least squares regression analysis comparing the absolute values of the standardized PIC values versus their standard deviations (see Garland et al. 1992). These regression lines did not differ significantly from zero (dichromatism: *F*_1,18_ = 2.522, *R*^2^ = 0.123, *P* = 0.130; latitude: *F*_1,18_ = 2.629, *R*^2^ = 0.127, *P* = 0.122), indicating that our tree and model of evolution adequately fit our data. We then tested for a relationship between contrasts of the two traits using linear regression forced through the origin.

## Results

### Plumage evolution in males and females

Although, in total, more plumage changes were reconstructed in males (146 changes) than in females (135 changes), the mean number of changes in each plumage character on the tree did not differ between the sexes (males: 4.87 ± 0.41; females: 4.5 ± 0.52; paired *t*-test; *t*_29_ = 0.73; *P* = 0.471). Furthermore, male and female plumage characters showed remarkably similar scores for overall CI (males = 0.56; females = 0.56) and overall RI (males = 0.59; females = 0.48), indicating that levels of evolutionary convergence in male plumage and female plumage were similar.

**Figure 2:**
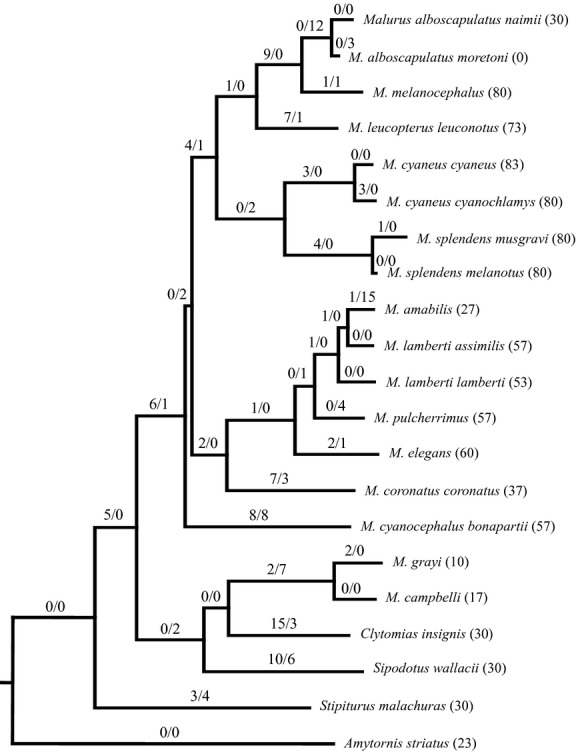
Evolutionary changes in plumage characters (indicated for males/females above each branch), measured as the number of unambiguous character state changes in each sex on the molecular phylogeny (from Driskell et al. 2011). Numbers in parentheses beside taxon names are levels of sexual dichromatism, measured as the percentage (%) of plumage character differences between males and females within each taxon. Dichromatism has decreased twice in *Malurus* due to dramatic changes in female colors: once in the ancestor of *Malurus amabilis* and once in the ancestor of *M. alboscapulatus naimii* and *M*. *a*. *moretoni*. Branch lengths on the tree reflect molecular changes.

Reconstructing ancestral color changes in each sex on the molecular phylogeny showed that changes in plumage colors have occurred in both sexes throughout the history of the Maluridae (Fig. 2; character changes in males/females shown above branches). In some taxa, such as the blue group (*M. cyaneus* and *M. splendens*), nearly all plumage changes have occurred in males and few have occurred in females. In contrast, in other groups, such as the chestnut-shouldered group (*M. elegans*, *M. lamberti*, *M. pulcherrimus*, and *M. amabilis*), the greatest number of changes have occurred in females. On 12 branches of the phylogeny (30% of total branches), unambiguous changes occurred in both sexes rather than just one or the other, whereas no unambiguous changes were reconstructed on nine (22.5%) of the branches. Reconstructing our plumage characters using relationships proposed by Lee et al. (2012) altered the number of male/female character changes in *M. coronatus coronatus* from 7/3 to 4/2, and changes on two deeper branches differed by only one of 30 plumage characters. Otherwise, however, the overall patterns of change using either phylogeny were identical.

Some of the most dramatic color changes have occurred in females, which in turn have affected levels of dichromatism. For example, in *M. amabilis* (Fig. 1D), 15 (50%) plumage characters changed in females, whereas only one character changed in males, resulting in much lower levels of dichromatism in this species (27%) than in closely related taxa (>53% in *M. lamberti*, *M. pulcherrimus*, and *M. elegans*; Fig. 2). Likewise, 12 (40%) of female plumage characters changed in the ancestor of *M. alboscapulatus* (Fig. 1E) and another three (10%) later changed in the subspecies *M. alboscapulatus moretoni*, whereas no plumage changes occurred at all in males. This subspecies was the only taxon in our study in which males and females exhibited no differences in plumage (Fig. 2), and this monochromatism appears to be entirely due to recent, rapid color changes in females.

**Figure 3:**
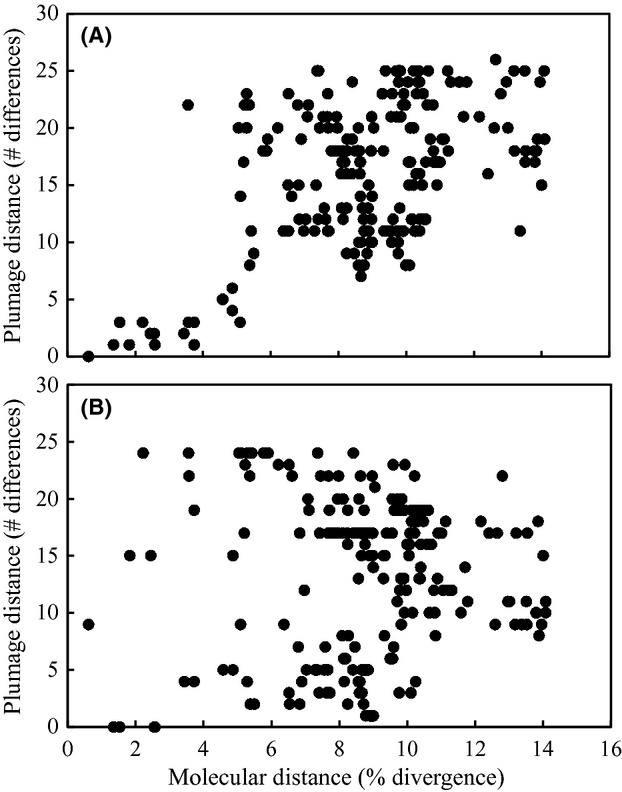
Plots showing pair-wise plumage distances between taxa as a function of molecular sequence divergence in (A) males and (B) females. Male plumage differences have accumulated steadily and almost linearly as a function of molecular distance between taxa (*R*^2^ = 0.262), whereas female plumage distances show no clear relationship with molecular distance (*R*^2^ = 0.005). Molecular divergence values are uncorrected *p* distances based on four mitochondrial and three nuclear regions.

In plots of plumage distance versus molecular sequence divergence, males and females showed very different evolutionary patterns. These pair-wise comparisons were not corrected for phylogeny and so should be interpreted with caution, but they nevertheless reveal some striking differences between the sexes. Male plumage differences appear to have accumulated almost linearly as a function of molecular distance between taxa (Fig. 3A; *R*^2^ = 0.262; analysis of variance [ANOVA]: *F*_1,208_ = 73.947; *P* < 0.001), suggesting that male colors have diverged at a relatively constant rate during the evolutionary history of the clade and that male color similarity among taxa is a reasonably accurate indicator of the genetic distance between them. In contrast, female plumage distance shows no clear relationship with molecular distance (Fig. 3B; *R*^2^ = 0.005, *F*_1,208_ = 0.971, *P* = 0.326). Female plumage patterns have diverged much more rapidly than male plumage in some cases and much less rapidly in others, suggesting widely varying rates of plumage evolution. Thus, although levels of evolutionary convergence in male plumage and female plumage are not appreciably different, female color patterns provide relatively little information about relationships among taxa.

### Fitting the evolutionary models

**Figure 4:**
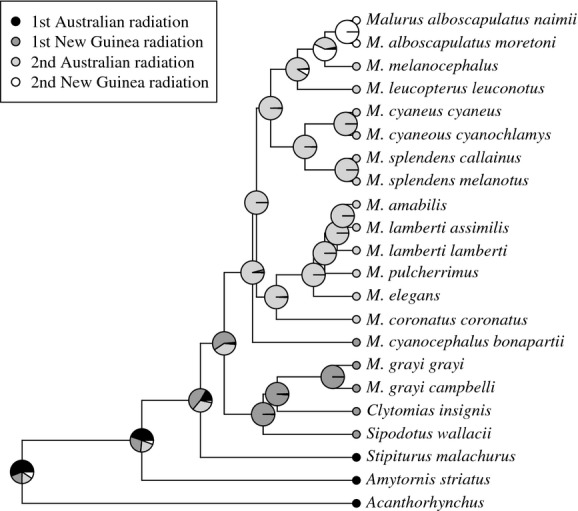
Ancestral reconstruction of geographic state of Maluridae, using radiations described in Schodde (1982). Pie charts on nodes represent the probability of each state being the resolved ancestral state for that node. The category first Australian radiation is resolved as the ancestral state for the entire tree, with a probability of 60.4%.

In line with findings above, plumage color changes in male and female Malurids corresponded to different evolutionary models. The best fitting evolutionary model for male plumage changes was BM, and the best fitting model for female changes was a multioptimum OU model with two adaptive peaks (Table 1). For males, the Delta AIC for the BM model was two or more points below each of the other three models making this the best fitting model. The three other models (single-peak OU, two-peak OU, and four-peak OU) could not be distinguished from one another. For females, the Delta AIC value of the two-peak OU model was approximately four points below the next best model (four-peak OU) and more than 20 points below the other two models, making this the best model. Based on this two-peak model, with initial states being either Australia or New Guinea, Australia had a 70.51% likelihood of being the ancestral point of radiation for the Maluridae. For the four-peak model, in which adaptive peaks corresponded to four historical distributions (Schodde 1982), the first category (Australian distribution of emu-wrens and grasswrens) had a 60.39% likelihood of being the ancestral state for the family (Fig. 4). Both OU models support the general hypotheses of Schodde (1982) that the family first evolved in Australia and that the genus *Malurus* initially evolved in New Guinea.

Our reconstruction of ancestral geography using character states from the four-peak model indicated multiple historical movements of malurid lineages between Australia and New Guinea (Fig. 4). This reconstruction suggested that all Australian fairy-wrens originated from New Guinea ancestors and that at least one New Guinea fairy-wren, *M. alboscapulatus*, was derived from a subsequent dispersal event back to New Guinea. Our log-likelihood analysis also suggested that another New Guinea fairy-wren, *M. cyanocephalus*, originated from Australian ancestors (Fig. 4), although the distant relationships between this species and other *Malurus* taxa make the timing of this event less clear.

### Relating dichromatism to latitude

**Figure 5:**
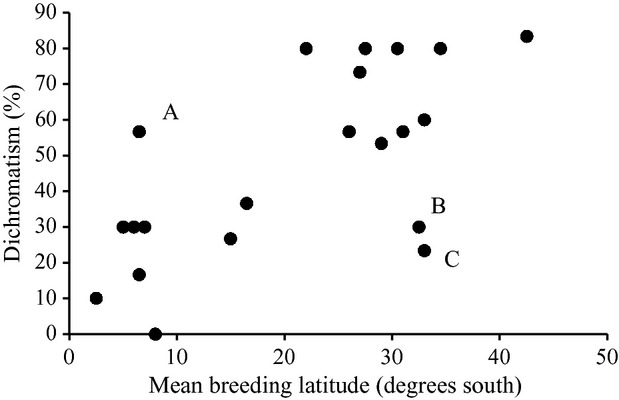
Pair-wise comparisons between dichromatism (percentage of plumage characters that differ between the sexes) and mean breeding latitude (degrees south, scored as the mid-point between northern and southern latitudinal limits of distribution) for 21 malurid taxa. Levels of dichromatism are significantly higher at more southerly latitudes in a phylogenetically independent contrasts analysis (ANOVA: *F*_1,19_ = 8.07, *R*^2^ = 0.298, *P* = 0.010). Points representing (A) *Malurus cyanocephalus*, (B) the emu-wren *Stipiturus malachurus*, and (C) the grasswren *Amytornis striatus* are indicated.

A PIC analysis comparing dichromatism (% of plumage character differences between the sexes) to mean breeding latitude revealed a significant positive relationship (ANOVA: *F*_1,19_ = 8.07, *R*^2^ = 0.298, *P* = 0.010; Fig. 5). This relationship was even stronger when we compared dichromatism to the lower latitudinal limit of each taxon's range (*F*_1,19_ = 16.053, *R*^2^ = 0.458, *P* = 0.001). The farther south a taxon's geographical distribution extended, the more dichromatic were the males and females in that taxon.

Three species provide interesting exceptions to the otherwise strong relationship between dichromatism and latitude in this group. The fairy-wren *M. cyanocephalus*, which appears to have dispersed from Australia to New Guinea (Fig. 4), is much more dichromatic than any other New Guinea fairy-wren (Point A in Fig. 5). Conversely, the emu-wren (*Stiptiturus*) and grasswren (*Amytornis*) species included in our study are both notably less dichromatic than Australian *Malurus* taxa found at the same latitudes (Points B and C, respectively, in Fig. 5).

## Discussion

For a relatively small family, the Maluridae shows remarkable variation in plumage dichromatism. There are comparatively monochromatic species in which both males and females are relatively dull (emu-wrens) or relatively bright and conspicuous (New Guinea species of *Malurus*), and dichromatic species in which males are brighter than females (most *Malurus*) (Schodde 1982; Rowley and Russell 1997). Furthermore, at least one species (*M. alboscapulatus* in New Guinea) shows almost the entire range of possible levels of plumage dichromatism. In this species, males are invariant in their plumage (glossy black and white), whereas female plumage varies considerably across six populations. In one population (*M. alboscapulatus lorentzi*), females are dull brown; in two populations (*M. a. alboscapulatus* and *M. a. naimi*), females are pied in coloration (brown, black, and white); and in three populations (*M. a. aida*, *M. a.kutubu*, and *M. a. moretoni*), females are black and white, identical in plumage to males (Rowley and Russell 1997). This variation in plumage is similar to variation in other bird species in which plumage coloration is linked to aspects of biology such as mating system or breeding latitude (Prum 1990, 1997; Friedman et al. 2009; Price and Whalen 2009), making plumage variation in fairy-wrens potentially interesting.

Here, we show that plumage characters in both males and females of species in Maluridae show similar overall numbers of changes during the evolutionary history of the group. Nevertheless, in some groups most plumage changes have occurred in males (e.g., the blue group), whereas in other groups most plumage changes have occurred in females (e.g., the chestnut-shouldered group). Importantly, these patterns of evolutionary change in plumage characters were similar regardless of whether we utilized the phylogeny of Driskell et al. (2011) or that of Lee et al. (2012).

Despite the fact that males and females show similar numbers of plumage changes and levels of homoplasy, the sexes nevertheless exhibited very different patterns of plumage evolution across the family. Differences between the plumage colors of males correlated well with genetic distance between species, whereas no such relationship existed for females (Fig. 3). This difference between the sexes was further emphasized by the results of the evolutionary models analysis, which indicated that plumage changes in males have accumulated steadily over time according to a BM model. Plumage changes in females, in contrast, best fit an OU model indicating natural selection, specifically a multioptimum model with two adaptive peaks corresponding to New Guinea and Australia.

The steady divergence of male plumage colors in the Maluridae is strikingly similar to patterns of male secondary sexual trait evolution found in phylogenetic studies of some other avian clades. In the oropendolas (family Icteridae), for instance, both male plumage colors and male song characteristics have diverged along with genetic distance among taxa at surprisingly regular rates, with almost no examples of evolutionary convergence in these traits (Price and Lanyon 2002; Price and Whalen 2009). Likewise, in the manakins (family Pipridae), male plumage traits and display elements show similar patterns of divergence and low levels of homoplasy (Prum 1990, 1997). Both of these groups are highly polygynous and, like fairy-wrens, are presumably strongly influenced by sexual selection. However, why male traits in these taxa should show such remarkably constant and cumulative patterns of evolution remains unclear (but see Prum 2010 for an intriguing explanation).

Unlike the steady divergence of male plumage patterns over time, female colors appear to have changed relatively little in some lineages and relatively dramatically in others, often resulting in large changes in dichromatism. For example, in both *M. amabilis* and *M. alboscapulatus*, females independently gained bright coloration in their plumages and now look more like males than do the females of closely related taxa. In fact, in one subspecies of *M. alboscapulatus*, *M. a. moretoni*, males and females were identical in all plumage scores in our study. Both of these species have relatively northerly distributions in comparison to closely related taxa (Rowley and Russell 1997), so these rapid decreases in dichromatism appear to have occurred along with movements from higher to lower (more northern) latitudes.

Our results suggest that, during the history of the Maluridae, selection on plumage of females has been very different in New Guinea and Australia. This is corroborated by the positive correlation between percent dichromatism and breeding latitude. Dichromatism is generally greater in Australian species than it is in New Guinea species, and within Australia the species distributed farther south are more dichromatic than those species in northern regions. Moreover, all male fairy-wrens are brightly colored, whereas females tend to have male-like plumage in the north and duller, more cryptic colors in the south (Schodde 1982; Rowley and Russell 1997). The one exception to this pattern among the fairy-wrens, the New Guinea species *M. cyanocephalus*, has relatively high levels of dichromatism more similar to Australian congeners than to other New Guinea taxa (Fig. 5). This species also appears to have dispersed from Australia, based on our reconstruction of ancestral geographic ranges (Fig. 4), which suggests that its high levels of dichromatism may be a retained ancestral state.

Two dramatic and related differences between the environments of New Guinea and Australia might relate to the latitudinal differences we observe in dichromatism. First, moving south into Australia from New Guinea, there is the gradient from tropical forests, to temperate forests, to savannah, and finally to open grasslands (Rowley and Russell 1997). Second, there is the gradient toward greater environmental seasonality moving south. This occurs both in comparing New Guinea to Australia and also within the continent of Australia. Whether it is habitat structure, seasonality, or a combination of the two that has yielded selection pressures on females for different levels of crypsis remains unknown. Nevertheless, we suggest that it is directly or indirectly related to predation at nests and the advantages of crypsis for incubating females (Martin and Badyaev 1996).

Interestingly, emu-wrens and grasswrens differ from this general latitudinal pattern in that both males and females in these species are dull colored and less dichromatic than fairy-wrens at similar latitudes (Fig. 5). Evidence suggests that these taxa also have much lower rates of extra-pair copulation than do Australian fairy-wrens (Rowe and Pruett-Jones 2013), which may explain the lack of bright colors in males (Webster et al. 2007). However, the potential influences of sexual selection and ecological factors in explaining these relatively low levels of dichromatism remain to be investigated, perhaps in comparative studies including additional emu-wren and grasswren taxa.

In a study similar to ours, but focusing primarily on female fairy-wrens, Karubian (2013) scored plumage ornamentation as high, moderate, or low and also showed that female ornamentation increased in species whose distributions were closer to the equator (lower latitudes). In keeping with our results that plumage changes in female malurids fit an evolutionary model indicating selection, Karubian (2013) also argues that plumage ornamentation in females is likely the result of selection rather genetic correlations with males or other factors. Karubian (2013) also examines variation in bill coloration across fairy-wrens and shows that it is substantially more variable in females than in males, and that, as with plumage, some of the variation is associated with geography (tropical species have darker bills than species in temperate areas).

Our result that dichromatism varied with latitude adds to an existing literature showing that patterns of dichromatism vary between tropical and temperate regions (Hamilton 1961; Price and Birch 1996; Friedman et al. 2009). In these previous studies, however, it was not latitude per se that was the focus of the association, but rather migratory behavior of the species. For example, using phylogenetic comparative methods, Friedman et al. (2009) reported a statistically significant association between the evolution of dichromatism and migratory behavior in New World orioles (*Icterus*), due to losses of bright plumage in the females of migratory, temperate breeding taxa. Our data show similar patterns in *Malurus*, in which different levels of dichromatism occur at different latitudes, largely due to changes in female plumage. Nevertheless, fairy-wrens are not migratory, and thus selection associated with migration cannot explain our findings. In both fairy-wrens and orioles, relatively dichromatic species occupy habitats with greater seasonality and relatively monochromatic species are found in the tropics (Rowley and Russell 1997; Jaramillo and Burke 1999). Friedman et al. (2009) argue that elaborate plumage in females may be advantageous in sedentary species and possibly maladaptive in migratory species. However, it could be that differences in female oriole plumage are better explained by differences between tropical and temperate habitats. These alternatives cannot be distinguished in the oriole group, in which all temperate breeding species are also migratory (Jaramillo and Burke 1999). Thus, our data for fairy-wrens suggest that migration per se may not be as important in plumage evolution as Friedman et al. (2009) suggest.

In this study, we visually scored the plumages of males and females of each species using methods similar to those used in other recent studies of color evolution (Irwin 1994; Burns 1998; Omland and Lanyon 2000; Price and Whalen 2009). Nevertheless, it is now well known that ultraviolet light is important in the plumage and vision of birds (Cuthill et al. 2000). In a recent study, Eaton (2005) reports that >90% of 139 presumably monochromatic species are, in fact, dichromatic based on avian vision and plumage reflectance data. These results suggest that most “visually” monochromatic bird species are likely dichromatic as perceived by birds (Eaton 2005) and argue that many of our standard paradigms concerning avian plumage evolution may be incorrect. We did not assess ultraviolet reflectance in our study, and we acknowledge that some of the species we categorized as relatively monochromatic may be more dichromatic than our subjective scores indicate. Nevertheless, our measures presumably reflect the wide range in relative levels of dichromatism characteristic of the Maluridae.

Plumage colors in the Maluridae, as in any bird family, are a result of complex interactions between evolutionary history, ecology, and sexual selection (Bennett and Owens 2002; Badyaev and Hill 2003). It is clear, however, that regardless of selection pressures affecting males (e.g., sexual selection), changes in female plumage are equally important in generating current patterns of dichromatism. Our evolutionary models analyses suggest that males and females in the Maluridae are often under different selective pressures and that these have had significant impacts on patterns of species divergence. Our results strengthen previous suggestions (Irwin 1994; Wiens 2001; Hofmann et al. 2008; Friedman et al. 2009) that when researchers are studying dichromatic species in which males are bright and females are dull, they should not immediately assume that this is the result of sexual selection in males. In fairy-wrens, we know that sexual selection represents a significant selective pressure on traits in males (Rowe and Pruett-Jones 2006, 2011, 2013; Webster et al. 2007), but sexual selection does not, in fact, appear to be solely responsible for the observed patterns of dichromatism in *Malurus*.

More broadly, our results highlight the importance of incorporating models of evolution with phylogenetic analyses of present and past character states. Our phylogenetic analyses, by themselves, showed that plumage in both males and females in malurid species were changing at approximately the same rates on average, but it was the evolutionary models analysis that indicated it was females that were more often under stronger directional selection, either for increased or decreased plumage elaboration. With the increasing ease of incorporating such evolutionary models analyses in research, we believe that future studies will confirm the importance of selection on plumage in females as a major force underlying existing patterns of dichromatism in birds.

## Appendix

**Table A1:** Plumage scores for male fairy-wren, grasswren, and emu-wren (Maluridae) species, as well as *Acanthorhynchus tenuirostris*

	Crown	Black Crown Spot	Supercillium	Superloral	Eye-line	Eye-ring	Lores
*Malurus splendens melanotus*	B	0	B	B	1	B	N
*M. splendens musgravi*	B	0	B	B	1	B	N
*M. coronatus coronatus*	B	1	B	B	1	N	N
*M. cyaneus cyaneus*	B	0	B	B	1	B	N
*M. cyaneous cyanochlamys*	B	0	B	B	0	B	N
*M. lamberti lamberti*	B	0	B	B	0	B	N
*M. lamberti assimilis*	B	0	B	B	0	B	N
*M. pulcherrimus*	B	0	B	B	0	B	N
*M. amabilis*	B	0	B	B	0	B	N
*M. elegans*	B	0	B	B	0	B	N
*M. leucopterus leuconotus*	B	0	B	B	0	B	B
*M. melanocephalus*	N	0	N	N	0	N	N
*M. alboscapulatus naimii*	N	0	N	N	0	N	N
*M. alboscapulatus moretoni*	N	0	N	N	0	N	N
*M. cyanocephalus bonapartii*	B	0	N	N	1	N	N
*Sipodotus wallacii*	N	0	N	N	1	W	N
*Clytomias insignis*	C	0	C	C	0	C	C
*M. grayi grayi*	N	0	B	B	1	B	N
*M. campbelli*	N	0	B	B	1	B	N
*Stipiturus malachurus*	C	0	B	C	0	W	C
*Amytornis striatus*	C	0	C	C	0	W	C
*Acanthorhynchus tenuirostris*	N	0	N	N	1	N	N

	Aricular	Throat	Malar	Breast	Upper belly	Lower belly	Nape
*Malurus splendens melanotus*	B	B	N	N	B	W	N
*M. splendens musgravi*	B	B	N	N	B	B	N
*M. coronatus coronatus*	N	W	W	W	W	W	N
*M. cyaneus cyaneus*	B	N	N	N	W	W	N
*M. cyaneous cyanochlamys*	B	N	N	N	W	W	N
*M. lamberti lamberti*	B	N	N	N	W	W	N
*M. lamberti assimilis*	B	N	N	N	W	W	N
*M. pulcherrimus*	B	B	N	N	W	W	N
*M. amabilis*	B	N	N	N	W	W	N
*M. elegans*	B	B	N	N	W	W	N
*M. leucopterus leuconotus*	B	B	B	B	B	B	B
*M. melanocephalus*	N	N	N	N	N	N	N
*M. alboscapulatus naimii*	N	N	N	N	N	N	N
*M. alboscapulatus moretoni*	N	N	N	N	N	N	N
*M. cyanocephalus bonapartii*	N	B	B	B	B	B	N
*Sipodotus wallacii*	W	W	N	W	W	W	C
*Clytomias insignis*	C	C	C	C	C	C	C
*M. grayi grayi*	B	B	N	B	B	B	N
*M. campbelli*	B	B	N	B	B	B	N
*Stipiturus malachurus*	C	B	C	B	L	W	D
*Amytornis striatus*	D	W	N	L	L	L	C
*Acanthorhynchus tenuirostris*	N	C	W	W	N	L	C

	Scapulars	Mantel	Back	Tail	Tail edge	Wing coverts	Wing covert edges
*Malurus splendens melanotus*	N	B	N	B	W	B	B
*M. splendens musgravi*	N	B	N	B	W	B	B
*M. coronatus coronatus*	D	D	D	B	B	D	D
*M. cyaneus cyaneus*	N	B	N	B	B	N	N
*M. cyaneous cyanochlamys*	N	B	N	N	N	N	N
*M. lamberti lamberti*	C	B	N	B	B	D	D
*M. lamberti assimilis*	C	B	N	B	B	D	D
*M. pulcherrimus*	C	B	N	B	B	D	D
*M. amabilis*	C	B	N	B	W	D	D
*M. elegans*	C	B	N	B	B	D	D
*M. leucopterus leuconotus*	W	B	B	B	B	W	W
*M. melanocephalus*	C	C	C	N	N	N	N
*M. alboscapulatus naimii*	W	N	N	N	N	N	N
*M. alboscapulatus moretoni*	W	N	N	N	N	N	N
*M. cyanocephalus bonapartii*	B	B	N	B	B	B	B
*Sipodotus wallacii*	C	C	C	D	W	D	W
*Clytomias insignis*	D	D	D	C	C	C	C
*M. grayi grayi*	B	B	B	D	D	D	D
*M. campbelli*	D	D	B	D	D	D	D
*Stipiturus malachurus*	D	D	D	D	D	D	D
*Amytornis striatus*	C	C	C	D	D	D	D
*Acanthorhynchus tenuirostris*	D	D	D	D	W	D	D

	Secondary	Secondary tip	Primary	Beak	Flanks	Iridescence	Crown streaked blue
*Malurus splendens melanotus*	B	B	B	N	B	1	0
*M. splendens musgravi*	B	B	B	N	B	1	0
*M. coronatus coronatus*	D	D	D	N	L	1	0
*M. cyaneus cyaneus*	N	N	N	N	W	1	0
*M. cyaneous cyanochlamys*	N	N	N	N	W	1	0
*M. lamberti lamberti*	D	D	D	N	L	1	0
*M. lamberti assimilis*	D	D	D	N	W	1	0
*M. pulcherrimus*	D	D	D	N	L	1	0
*M. amabilis*	D	D	D	N	W	1	0
*M. elegans*	C	C	D	N	L	1	0
*M. leucopterus leuconotus*	W	W	D	N	B	1	0
*M. melanocephalus*	N	N	N	N	N	0	0
*M. alboscapulatus naimii*	N	N	N	N	N	0	0
*M. alboscapulatus moretoni*	N	N	N	N	N	0	0
*M. cyanocephalus bonapartii*	B	B	N	N	B	1	0
*Sipodotus wallacii*	D	D	D	N	W	0	1
*Clytomias insignis*	C	C	C	N	C	0	0
*M. grayi grayi*	D	D	D	N	B	1	0
*M. campbelli*	D	D	D	N	B	1	0
*Stipiturus malachurus*	D	D	D	N	L	0	0
*Amytornis striatus*	D	D	D	N	L	0	0
*Acanthorhynchus tenuirostris*	D	D	D	N	L	0	0

	White streaking	Black streaking
*Malurus splendens melanotus*	0	0
*M. splendens musgravi*	0	0
*M. coronatus coronatus*	0	0
*M. cyaneus cyaneus*	0	0
*M. cyaneous cyanochlamys*	0	0
*M. lamberti lamberti*	0	0
*M. lamberti assimilis*	0	0
*M. pulcherrimus*	0	0
*M. amabilis*	0	0
*M. elegans*	0	0
*M. leucopterus leuconotus*	0	0
*M. melanocephalus*	0	0
*M. alboscapulatus naimii*	0	0
*M. alboscapulatus moretoni*	0	0
*M. cyanocephalus bonapartii*	0	0
*Sipodotus wallacii*	0	0
*Clytomias insignis*	0	0
*M. grayi grayi*	0	0
*M. campbelli*	0	0
*Stipiturus malachurus*	1	1
*Amytornis striatus*	1	0
*Acanthorhynchus tenuirostris*	0	0

The ordering of taxa follows Rowley and Russell (1997). Color character state definitions are as follows: B, blue; C, carotenoid; N, black; W, white; D, dark brown; L, light brown. Other character states are as follows: 0, absent; 1, present.

**Table A2:** Plumage scores for female fairy-wren, grasswren, and emu-wren species (Maluridae), as well as *Acanthorhynchus tenuirostris*

	Crown	Black Crown Spot	Supercillium	Superloral	Eye-line	Eye-ring	Lores
*Malurus splendens melanotus*	D	0	D	D	0	C	C
*M. splendens musgravi*	D	0	D	D	0	C	C
*M. coronatus coronatus*	D	0	L	D	0	W	D
*M. cyaneus cyaneus*	D	0	D	D	0	C	C
*M. cyaneous cyanochlamys*	D	0	D	D	0	C	C
*M. lamberti lamberti*	D	0	D	D	0	C	C
*M. lamberti assimilis*	D	0	D	D	0	C	C
*M. pulcherrimus*	D	0	D	D	0	C	C
*M. amabilis*	B	0	B	B	0	W	W
*M. elegans*	D	0	D	D	0	D	C
*M. leucopterus leuconotus*	D	0	D	D	0	D	D
*M. melanocephalus*	D	0	D	D	0	L	L
*M. alboscapulatus naimii*	N	0	N	W	0	W	N
*M. alboscapulatus moretoni*	N	0	N	N	0	N	N
*M. cyanocephalus bonapartii*	B	0	N	N	0	N	N
*Sipodotus wallacii*	N	0	N	N	0	W	W
*Clytomias insignis*	C	0	C	C	0	C	C
*M. grayi grayi*	N	0	B	B	0	B	N
*M. campbelli*	N	0	B	B	0	B	N
*Stipiturus malachurus*	D	0	D	D	0	W	L
*Amytornis striatus*	D	0	D	C	0	W	C
*Acanthorhynchus tenuirostris*	D	0	D	D	0	N	D

	Aricular	Throat	Malar	Breast	Upper belly	Lower belly	Nape
*Malurus splendens melanotus*	L	W	W	W	W	L	D
*M. splendens musgravi*	L	W	W	W	W	L	D
*M. coronatus coronatus*	D	W	W	W	W	W	D
*M. cyaneus cyaneus*	L	W	W	W	W	L	D
*M. cyaneous cyanochlamys*	L	W	W	W	W	L	D
*M. lamberti lamberti*	D	W	W	W	W	L	D
*M. lamberti assimilis*	D	W	W	W	W	L	D
*M. pulcherrimus*	D	L	L	L	L	L	D
*M. amabilis*	B	W	W	W	W	W	B
*M. elegans*	D	W	W	W	W	L	D
*M. leucopterus leuconotus*	D	W	W	W	W	L	D
*M. melanocephalus*	D	W	W	W	W	L	D
*M. alboscapulatus naimii*	N	W	W	P	P	W	N
*M. alboscapulatus moretoni*	N	N	N	N	N	N	N
*M. cyanocephalus bonapartii*	N	B	N	W	W	L	B
*Sipodotus wallacii*	W	L	N	L	L	L	N
*Clytomias insignis*	C	L	C	C	C	L	C
*M. grayi grayi*	B	B	N	B	W	W	N
*M. campbelli*	B	B	N	B	W	W	N
*Stipiturus malachurus*	W	L	L	L	L	W	D
*Amytornis striatus*	D	W	N	L	L	L	D
*Acanthorhynchus tenuirostris*	D	D	D	L	L	L	C

	Scapulars	Mantel	Lower back	Tail	Tail edge	Wing coverts	Wing covert edges
*Malurus splendens melanotus*	D	D	D	B	B	D	D
*M. splendens musgravi*	D	D	D	B	B	D	D
*M. coronatus coronatus*	D	D	D	B	B	D	D
*M. cyaneus cyaneus*	D	D	D	D	D	D	D
*M. cyaneous cyanochlamys*	D	D	D	D	D	D	D
*M. lamberti lamberti*	D	D	D	B	B	D	D
*M. lamberti assimilis*	D	D	D	B	B	D	D
*M. pulcherrimus*	D	D	D	B	B	D	D
*M. amabilis*	B	B	B	B	W	D	D
*M. elegans*	C	D	D	D	D	D	D
*M. leucopterus leuconotus*	D	D	D	B	B	D	D
*M. melanocephalus*	D	D	D	D	D	D	D
*M. alboscapulatus naimii*	W	N	N	N	N	N	W
*M. alboscapulatus moretoni*	W	N	N	N	N	N	N
*M. cyanocephalus bonapartii*	C	C	C	N	W	D	D
*Sipodotus wallacii*	C	C	C	D	W	D	W
*Clytomias insignis*	D	D	D	D	D	D	D
*M. grayi grayi*	B	B	B	D	D	D	D
*M. campbelli*	B	B	B	D	D	D	D
*Stipiturus malachurus*	D	D	D	D	D	D	D
*Amytornis striatus*	D	D	D	D	D	D	D
*Acanthorhynchus tenuirostris*	D	D	D	D	D	D	D

	Secondary	Secondary tip	Primary	Beak	Flanks	Iridescence	Crown streaked blue
*Malurus splendens melanotus*	D	D	D	C	L	0	0
*M. splendens musgravi*	D	D	D	C	L	0	0
*M. coronatus coronatus*	D	D	D	N	L	0	0
*M. cyaneus cyaneus*	D	D	D	C	L	0	0
*M. cyaneous cyanochlamys*	D	D	D	C	L	0	0
*M. lamberti lamberti*	D	D	D	C	L	0	0
*M. lamberti assimilis*	D	D	D	C	L	0	0
*M. pulcherrimus*	D	D	D	C	L	0	0
*M. amabilis*	D	D	D	N	W	1	0
*M. elegans*	D	D	D	N	L	0	0
*M. leucopterus leuconotus*	D	D	D	C	L	0	0
*M. melanocephalus*	D	D	D	C	L	0	0
*M. alboscapulatus naimii*	N	W	N	N	N	0	0
*M. alboscapulatus moretoni*	N	N	N	N	N	0	0
*M. cyanocephalus bonapartii*	D	D	D	N	C	1	0
*Sipodotus wallacii*	D	D	D	N	L	0	1
*Clytomias insignis*	D	D	D	N	C	0	0
*M. grayi grayi*	D	D	D	N	B	1	0
*M. campbelli*	D	D	D	N	B	1	0
*Stipiturus malachurus*	D	D	D	N	L	0	0
*Amytornis striatus*	D	D	D	N	C	0	0
*Acanthorhynchus tenuirostris*	D	D	D	N	L	0	0

	White streaking	Black streaking
*Malurus splendens melanotus*	0	0
*M. splendens musgravi*	0	0
*M. coronatus coronatus*	0	0
*M. cyaneus cyaneus*	0	0
*M. cyaneous cyanochlamys*	0	0
*M. lamberti lamberti*	0	0
*M. lamberti assimilis*	0	0
*M. pulcherrimus*	0	0
*M. amabilis*	0	0
*M. elegans*	0	0
*M. leucopterus leuconotus*	0	0
*M. melanocephalus*	0	0
*M. alboscapulatus naimii*	0	0
*M. alboscapulatus moretoni*	0	0
*M. cyanocephalus bonapartii*	0	0
*Sipodotus wallacii*	0	0
*Clytomias insignis*	0	0
*M. grayi grayi*	0	0
*M. campbelli*	0	0
*Stipiturus malachurus*	0	1
*Amytornis striatus*	1	0
*Acanthorhynchus tenuirostris*	0	0

The ordering of taxa follows Rowley and Russell (1997). Color character state definitions are as follows: B, blue; C, carotenoid; N, black; W, white; D, dark brown; L, light brown. Other character states are as follows: 0, absent; 1, present.
